# Visualizing orbital and arterial wall involvement in arteritic anterior ischemic optic neuropathy with time-of-flight simultaneous [^18^F]FDG-PET/MRI: a novel supportive diagnostic imaging modality?

**DOI:** 10.1016/j.ajoc.2026.102685

**Published:** 2026-10-17

**Authors:** Andreas Worm Bendtsen, Jane Maestri Brittain, Jonathan Frederik Carlsen, Michael Stormly Hansen, Lene Terslev, Thomas Lund Andersen, Steffen Hamann

**Affiliations:** a Department of Ophthalmology, Copenhagen University Hospital - Rigshospitalet, Denmark; b Department of Clinical Physiology and Nuclear Medicine, Copenhagen University Hospital, Rigshospitalet, Denmark; c Department of Radiology, Copenhagen University Hospital - Rigshospitalet, Denmark; d Department of Rheumatology and Spine Diseases, Copenhagen University Hospital – Rigshospitalet, Denmark; e Department of Clinical Medicine, University of Copenhagen, Copenhagen, Denmark; f Rothschild Foundation Hospital, Paris, France

**Keywords:** A-AION, NA-AION, Orbital inflammation, FDG-PET/MRI

## 1 Case report

A 77-year-old woman presented with acute vision loss in the left eye with a best corrected visual acuity of 20/50, pronounced ipsilateral relative afferent pupillary defect, optic disc edema, and an adjacent cotton wool spot with underlying paracentral acute middle maculopathy (Fig. 1a–b). Systemic symptoms included jaw claudication and new-onset temporal headache. CRP was elevated to 37 mg/L. Color Doppler ultrasound demonstrated bilateral vasculitis of the temporal, facial, and vertebral arteries, and the patient was diagnosed with giant cell arteritis (GCA) and arteritic anterior ischemic optic neuropathy (A-AION). Corticosteroid treatment was initiated on presentation. After five days of treatment, a regional [^18^F]FDG-PET/MRI of the orbits and the cranial and cervical arteries was performed on a 3-T Biograph One (Siemens Healthineers) with an administered activity of 3 MBq/kg and 0.1 mmol/kg gadolinium-based contrast agent. Pathological FDG uptake in the vertebral and temporal arteries bilaterally, combined with marked mural enhancement of these vessels on post-contrast 3D fat-saturated T1 images with blood suppression (Fig. 2a), was consistent with GCA despite ongoing corticosteroid treatment.^1^, ^2^ Furthermore, enhancement of the retrobulbar and perineural fat along the posterior ciliary arteries (PCAs) (Fig. 2b) in the left orbit on post-contrast axial T1 fat-saturated images supported the diagnosis of ipsilateral A-AION due to PCA occlusion.^1^, ^2^

**Fig. 1:**
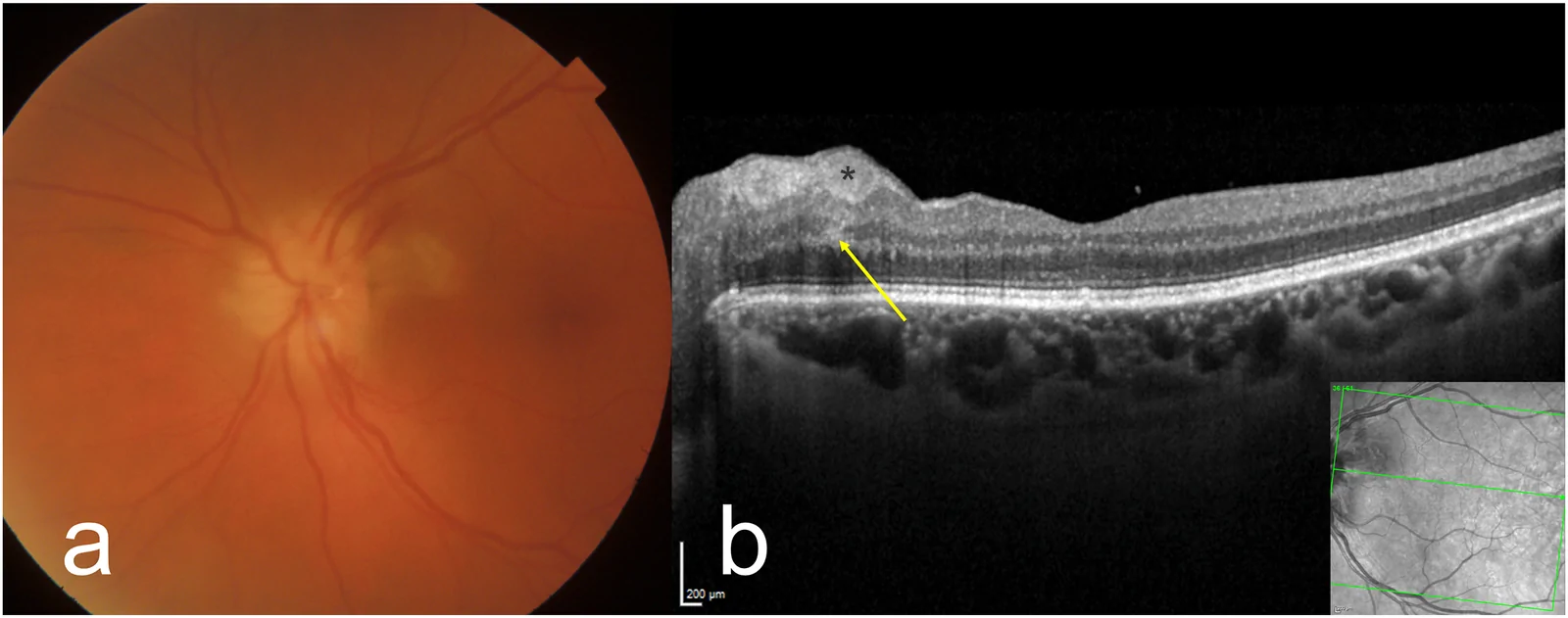
Left eye at presentation. **a:** Fundus photograph showing pallid optic disc edema and a cotton wool spot. **b:** Optical coherence tomography with cotton wool spot (*) and paracentral acute middle maculopathy (arrow).

**Fig. 2:**
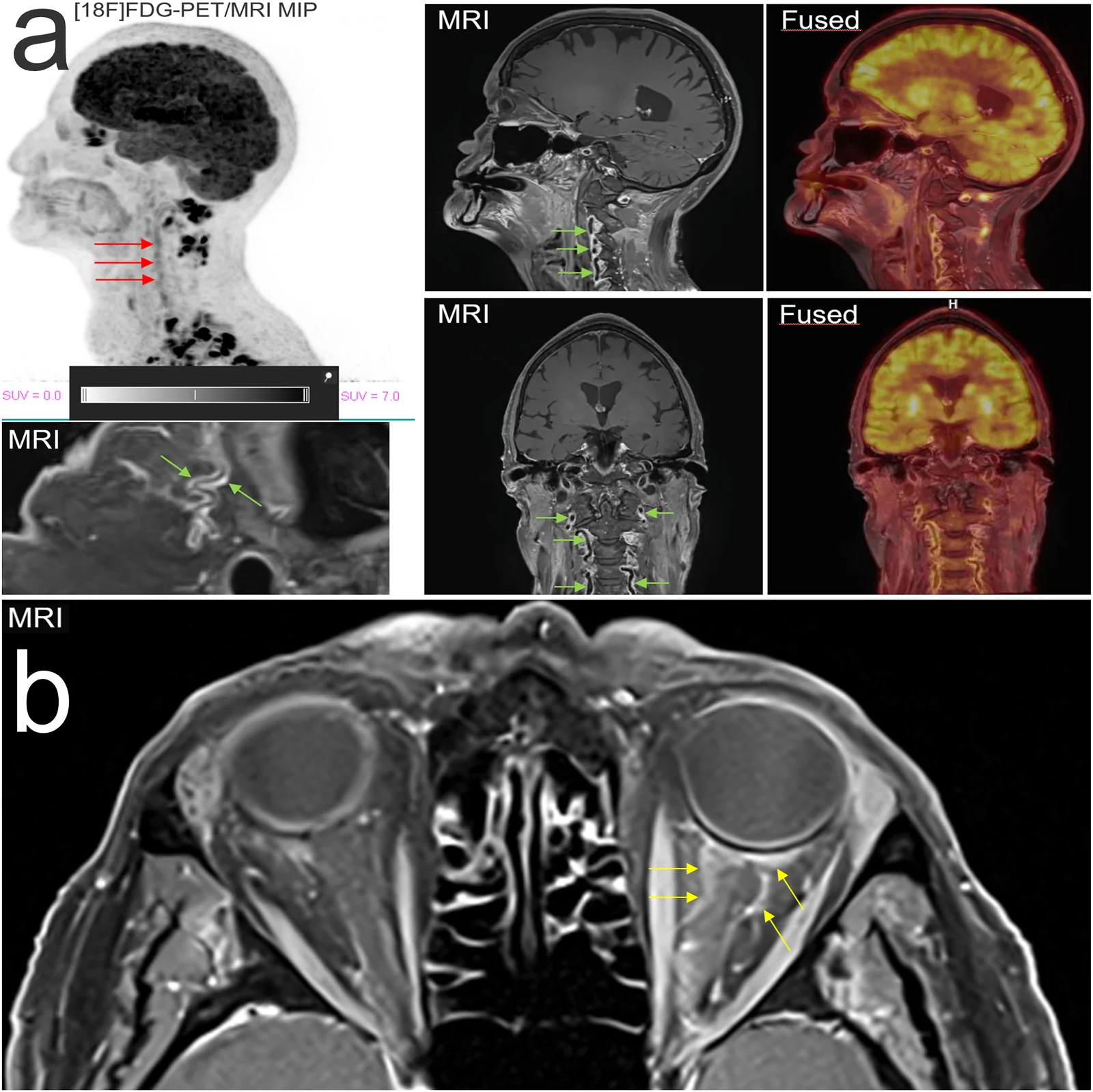
**a:** Representative [^18^F]FDG-PET, MRI and fused PET/MR images of cranial and cervical arteries. Increased FDG uptake is seen in the walls of both vertebral arteries on PET (red arrows), with wall thickening and contrast enhancement of the vertebral arteries and the left temporal artery (green arrows) on T1 MRI images with blood suppression, consistent with giant cell arteritis. **b:** Contrast enhancement of the perineural and the retrobulbar fat along the posterior ciliary arteries (yellow arrows) on T1 MRI images indicated periarterial orbital inflammation in arteritic anterior ischemic optic neuropathy. (For interpretation of the references to colour in this figure legend, the reader is referred to the Web version of this article.)

## 2 Discussion

Differentiating A-AION from non-arteritic AION can be clinically challenging but is critically important given treatment implications. Combining orbital and vessel wall MRI with [^18^F]FDG-PET offers simultaneous high-resolution structural and molecular imaging in a single examination. This multimodal approach has the potential to surpass the accuracy of current diagnostic modalities for evaluating GCA in patients presenting with AION, a hypothesis we are prospectively testing (ClinicalTrials.gov registry NCT07206238).

## 3 Conclusion

[^18^F]FDG-PET/MRI may demonstrate periarterial orbital inflammation together with cranial and cervical vasculitis, offering a comprehensive single-session evaluation. If validated prospectively, this approach could meaningfully improve differentiation of AION subtypes and guide early targeted therapy in GCA.

## CRediT authorship contribution statement

**Andreas Worm Bendtsen:** Writing – original draft, Visualization, Methodology, Investigation, Data curation, Conceptualization. **Jane Maestri Brittain:** Writing – original draft, Visualization, Methodology, Investigation, Formal analysis, Data curation, Conceptualization. **Jonathan Frederik Carlsen:** Writing – review & editing, Methodology, Formal analysis, Conceptualization. **Michael Stormly Hansen:** Writing – review & editing, Supervision, Methodology, Conceptualization. **Lene Terslev:** Writing – review & editing, Supervision, Methodology, Investigation, Conceptualization. **Thomas Lund Andersen:** Writing – review & editing, Methodology, Data curation, Conceptualization. **Steffen Hamann:** Writing – review & editing, Validation, Supervision, Project administration, Methodology, Funding acquisition, Conceptualization.

## Ethics declaration

Written informed consent to take part in the study and to publish the article has been obtained from all participants or their legal representatives. The privacy rights of participants have been observed.

This study was performed in compliance with relevant laws, regulatory frameworks and guidelines where the research took place. This study was approved by the Ethics Committee of the Capital Region of Denmark (Approval No. H-25043110). This study was performed in line with the principles of the Declaration of Helsinki.

This research follows the CARE guidelines and the CARE checklist.

## Declaration of generative AI use

Nothing to declare.

## Funding

This work was supported by The 10.13039/501100003554Lundbeck Foundation (R453-2024-308).

## Declaration of competing interest

The authors have nothing to disclose.
